# Higher Urinary Bisphenol A Concentration Is Associated with Unexplained Recurrent Miscarriage Risk: Evidence from a Case-Control Study in Eastern China

**DOI:** 10.1371/journal.pone.0127886

**Published:** 2015-05-26

**Authors:** Yueping Shen, Yanmin Zheng, Jingting Jiang, Yinmei Liu, Xiaoming Luo, Zongji Shen, Xin Chen, Yan Wang, Yiheng Dai, Jing Zhao, Hong Liang, Aimin Chen, Wei Yuan

**Affiliations:** 1 Jiangsu Key Laboratory of Preventive and Translational Medicine for Geriatric Diseases, Department of Epidemiology and Health Statistics School of Public Health, Soochow University, Suzhou, 215123, PR China; 2 Suzhou Center for Disease Prevention and Control, Suzhou 215004, China; 3 The Third Affiliated Hospital, Suzhou University, Changzhou 213003, China; 4 Department of Nosocomial Infection and Disease Control, Shanghai Tenth People’s Hospital, Shanghai 200072, China; 5 Maternal and Child Health Bureau of Kunshan, Kunshan 215301, China; 6 Department of Obstetrics and Gynaecology, First Affiliated Hospital of Soochow University, Suzhou 215006, China; 7 Department of Obstetrics and Gynaecology, Second Affiliated Hospital of Soochow University, Suzhou 215004, China; 8 School of Radiation Medicine and Protection, Medical College of Soochow University, Suzhou 215123, China; 9 Department of Reproductive Epidemiology and Social Science, Shanghai Institute of Planned Parenthood Research, Shanghai 200032, China; 10 Division of Epidemiology and Biostatistics, Department of Environmental Health, University of Cincinnati College of Medicine, Cincinnati, Ohio 45221, United States of America; 11 NPFPC Laboratory of Contraception and Devices, Shanghai 200032, China; 12 Institute of Reproduction & Development, Fudan University, Shanghai 200032, China; Deakin School of Medicine, AUSTRALIA

## Abstract

**Background:**

Evidence about the association between Bisphenol A (BPA) and the risk of recurrent miscarriage (RM) in human being is still limited.

**Objective:**

We evaluated the association of urinary BPA concentrations with RM in human being.

**Methods:**

A hospital-based 1:2 matched case-control study on RM was carried out in Suzhou and Kunshan in Jiangsu Province in China between August 2008 and November 2011. Total urinary BPA concentrations in 264 eligible urine samples (102 RM patients and 162 controls) were measured using liquid chromatography-tandem mass spectrometry (LC-MS/MS). The Wilcoxon test and conditional logistic regression were used to estimate the differences between the groups and odds ratios (OR) with 95% confidence intervals (CI), respectively.

**Results:**

The median ± *IQR* (interquartile range) (*P*_75_-*P*_25_) values of non-creatinine-adjusted total urinary BPA levels in the RM patients and the controls were 1.66±3.69ng/ml and 0.58±1.07ng/ml, respectively (0.98±2.67μg/g Cr (creatinine) and 0.40±0.77μg/g Cr. The adjusted BPA level was significantly higher in the RM patients than in the controls (Wilcoxon test, *Z* = 4.476, *P*<0.001). Higher level of urinary BPA was significantly associated with an increased risk of RM (*P*-trend <0.001). Compared to the groups with urinary BPA levels less than 0.16μg/g Cr, the women with levels of 0.40–0.93μg/g Cr and 0.93μg/g Cr or above had a significantly higher risk of RM (OR = 3.91, 95%CI: 1.23–12.45 and OR = 9.34, 95%CI: 3.06–28.44) that persisted after adjusting for confounding factors. The time from recently RM date to recruitment does not significantly influence the urinary BPA level (*P* = 0.090).

**Conclusion:**

Exposure to BPA may be associated with RM risk.

## Introduction

Recurrent miscarriage (RM) has traditionally been defined as three consecutive unexplained terminations of pregnancy before 20 weeks of gestation or expulsions of a fetus weighing<500g [1]. The prevalence of RM has been reported to be as high as 0.5–3% [2, 3]. There is a tendency to expand this definition to include women who have experienced only two miscarriages, particularly in studies focusing on the aetiology or risk factors of RM [1, 4]. The causes of RM are very complicated. The identifiable factors of RM may include parental chromosomal anomalies, uterine pathology, a prothrombotic state, endocrine disorders, immunological factors, and infections [5–10]. However, the causes of RM remain unexplained for approximately half of the women who experience RM, despite thorough investigations [4].

Currently, endocrine-disrupting chemicals (EDCs), including bisphenol A (BPA), polychlorinated biphenyls (PCBs), 1,1-dichloro-2,2-bis(p-chlorophenyl) ethylene (DDE) and hexachlorobenzene (HCB), are the most prominent potential causes of unexplained RM [11, 12]. BPA, a monomer used in the production of polycarbonates and epoxy resins and as an antioxidant in PVC plastics, is one of the most widely used industrial compounds worldwide. In general, polycarbonates are used to manufacture plastic food containers and the epoxy resins that coat the inner surfaces of food and beverage cans. PVC is used in a variety of products, including materials that may come into contact with food, such as plastic film used for packaging food. The migration of BPA from polycarbonate plastics, surfaces of epoxy-coated cans, and PVC products into food and food stimulants has been reported, especially when heated [13–15]. Humans may be exposed to BPA in food, beverages, dust, etc. BPA is a well-known endocrine disruptor with estrogenic and anti-androgen activities that can cause a variety of adverse effects in both animals and humans. Evidence from large experimental studies suggests that exposure to BPA has adverse effects on reproductive development in animals [16–21], and recently there two epidemiologic studies on the association between BPA exposure and miscarriage risk [22, 23], but human studies on the BPA associated with RM risk are still limited. Urinary BPA may be associated with lower semen quality, increased sperm DNA damage [24], and self-reported sexual dysfunction in males [25]. Bloom and colleagues assessed BPA in couples undergoing in vitro fertilization (IVF) and measured indicators of embryo quality. The study showed that male BPA exposure may affect embryo quality by influencing the early embryo cleavage rate during IVF [26]. Several studies found that serum BPA levels were associated with endometrial hyperplasia and ovarian dysfunction in females [27–29]. To date, only one study from Japan has reported findings about the relationship between serum BPA levels and RM [11].

We measured the total urinary BPA level (conjugated and free) to quantify the amount of human exposure to BPA. Although BPA has a short half-life in biological tissues [30, 31], urinary BPA is still considered to be the preferred indicator to assess biological exposure [32].

Suzhou and neighboring Kunshan cities are located in the lower reaches of the Yangtze River––the most industrialized areas in eastern China. After 30 years of rapid economic development and marked life style changes, environment challenges in this area on public health such as plasticizer use and other endocrine disrupting chemicals should be examined. The aim of the present study is to investigate the association between urinary BPA levels and unexplained RM, a relationship that has not yet been reported.

## Methods

### Subjects and recruitment

First, between August 2008 and November 2011, we initially registered 121 patients aged 20–40 years who sought treatment and self-reported history of RM with repeated (2–6 RMs), consecutive unexplained terminations of pregnancy before 20 weeks of gestation from the outpatients of obstetric and gynaecological clinics of three hospitals––the Maternal and Child Health Center in Kunshan City, the First People's Hospital, and the Second People's Hospital affiliated with Soochow University. The time from the nearest RM date for the patients to recruitment were various from 2 days to 352 days. The patients were asked to answer questionnaire, provide morning spot urine and blood samples less than two weeks after the recruitment. They also completed regular medical examination (B-mode ultrasound, autoimmune disorder test, and abnormal coagulate function test) to exclude other known causes of RM.

When the study was completed, 19 cases were excluded for the exclusion criteria including reproductive system malformation (uterus bicornis, uterus septus, uterine leiomyoma) (8), autoimmune disorders (anticardiolipin antibody- and antinuclear antibody-positive) (6) and abnormal coagulate function (5). We have not excluded the patients who suffered chromosome abnormalities and endocrine disorders. In each hospital, there are more than 20 pregnant women performing the routine prenatal examination in every working day, who were registered their name, age and gestational age, etc. Once acquired one eligible RM case, based on the prenatal examination name list, we tried to match 2 controls of pregnant women aged 20–40 years with no history of miscarriage in the same hospital with the following criteria: age (±2 years), gestational age (±1 week), and living in the same district. We excluded the controls who had a history of pregnancy complications, miscarriage, still birth or pre-eclampsia, and none had given birth to a small for gestational age infant. Totally 60 cases were successfully matched 2 controls, however 42 cases only had 1 match.

The controls were also asked to provide information on demographic characteristics, lifestyle, obstetric history, and other RM risk factors by a structured questionnaire and morning spot urine and blood samples. To avoid contamination from plastic equipment, glass containers were used in the sampling and experimental processes. The containers were soaked in nitric acid solution for at least 12h, washed with deionised water, and baked at 70°C for at least 10h. The urine samples were stored at -80°C before LC-MS/MS analysis. Written informed consent was obtained from each participant. The study was approved by the ethics committee of Soochow University.

### Analysis of Urinary BPA

LC-MS/MS was used to measure total BPA (conjugated and free) in the urine samples from the participants, using selective reaction monitoring (SRM). After pre-treatment, the samples (25μl) were separated by a BR-C18 column (5μm, 100×2.1mm; Sepax, USA) using a Thermo Finnigan high performance liquid chromatography system combined with a TSQ Quantum Ultra triple quadrupole tandem mass spectrometer (Thermo Electron, Waltham, MA, USA) equipped with an electrospray ionization probe (Thermo Electron). Gradient elution with a 0.3% ammonia solution (solvent A) and methanol (solvent B) was carried out using the following procedure: 40% B for 0.5min, gradually increased to 95% B within 2min, solvent composition held at 95% B for 1.5min and equilibrated at 40% B for 1.5min. The total chromatographic separation time was 5.5min. The flow rate was 300μl/min. To record spectral data, a vaporizer temperature of 300°C and a TurboIonSpray voltage of -3.0kV in the negative ionization mode were applied. Nitrogen served as the sheath gas, the auxiliary gas, and the ion sweep gas; the values of these gases were set to 50, 10, and 1 arbitrary unit(s), respectively. The mass spectrometer was operated in the selected reaction monitoring (SRM) mode with a signal time segment. The scan width for SRM was 0.01m/z, scan time was 0.1s, and the peak width settings (FWHM) for both Q1 and Q3 were 0.7.

The pre-treatment process was a modified version of the methods used by Matsumoto [33]. Urine (500μl) and 50μl d16-BPA 200ng/ml (internal standard) were buffered with 350μl of 1.0 M sodium acetate buffer (pH5.0) and hydrolysed enzymatically with β-glucuronidase/sulfatase(110,000U/ml/4,000U/ml, Sigma Chemical Co., USA) at 37°C overnight in a shaking water bath [34]. After hydrolysis, the hydrolysate was extracted once with 5ml of ethyl acetate. After centrifugation, the supernatant was transferred to a new glass tube and evaporated with N_2_ gas. The residue was dissolved with 500μl of 30/70 (v/v) methanol/water solution, filtered using a microporous membrane filter and injected into the LC-MS/MS system described above.

**Fig 1:**
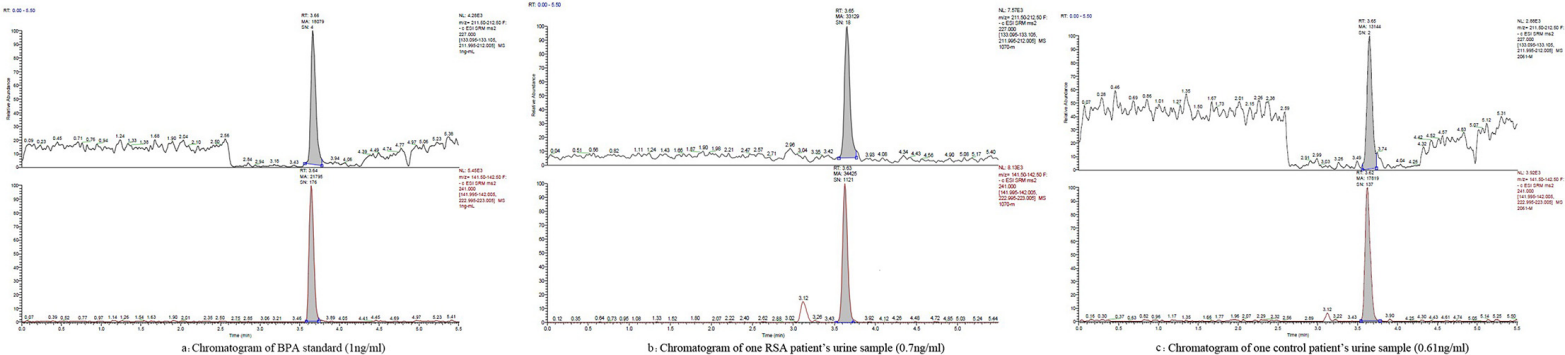
Chromatogram of BPA standard (1ng/ml) (a), one RM patient’s urine sample (0.7ng/ml) (b) and one control patient’s urine sample (0.61ng/ml) (c).

The standard curve of this method was y = 0.1304+0.6801x. Satisfactory linearity was obtained (r>0.999). The limit of detection (LOD) of urinary BPA was 0.20ng/ml, similar other studies [35, 36]. Recoveries of BPA ranged from 105.0% to 109.5%. The repeatability of the method was evaluated by analysing six replicates of four samples spiked with standard BPA. Expressed as the relative standard deviation (RSD) within each day and between days, the values were calculated as 3.16%-4.22% and 5.22%-5.30%, respectively. Fig 1 showed the chromatograms of standard, case and control urine samples, which had concentrations of 1ng/ml, 0.7ng/ml and 0.61ng/ml, respectively. BPA levels were measured by comparing the retention time to the retention time of the main peak (3.6min) in the chromatogram of standard BPA. To measure BPA concentration, a mean blank value of 0.4ng/ml had been determined and was subtracted from each measured value. In addition, the BPA value was adjusted for the urinary creatinine concentration to correct for the urine volume. Creatinine was detected using the method of measuring enzymatic creatinine in an automatic biochemical instrument (Hitachi 7020). The concentration of urinary BPA was expressed in micrograms of BPA per gram of creatinine.

### Statistical analysis

We imputed the missing levels by the value of the LOD divided by the square root of 2 if the geometric standard deviation (GSD) was less than 3; otherwise, we imputed by the LOD divided by 2 [35, 37]. We used the Wilcoxon method to compare the difference in urinary BPA levels between RM patients and controls or between other different groups because the urinary BPA levels of the patients in the two groups were not normally distributed. Univariate and multivariate conditional logistic regression were used to calculate crude and adjusted ORs (95%CI), respectively, to estimate the association between urinary BPA and RM. Potential confounders include age, occupation, years of education, BMI and passive smoking during pregnancy in our study. Statistical power was calculated using SAS Proc power. On the basis of prevalence of urinary BPA ≤0.16 μg/g Cr in 25% of general women population and a ratio of two control subjects to 1 case, 102 cases and 162 control subjects has 92.7% power to detect an odds ratio of 2.5. The significance level was set at *P*<0.05 for all tests. SAS 9.2 software (SAS Institute, Inc., Cary, NC) was used for all analyses.

## Results

### Characteristics of subjects

The mean age ± SD of the RM patients was 28.04±3.68 years, and the mean age of the controls was 28.36±3.75 years. On average, the women in the case group had experienced 2.47 previous miscarriages (2 to 6 RMs), some consecutively and some not consecutively but at least 2 consecutive. There were no significant differences in age or occupation between the case and control groups (*P*>0.05). However, BMI on average was significantly lower among the controls than among the RM patients (*P*<0.001). The distributions of BMI and the years of education of the two groups were significantly different (*P* = 0.047 and *P* = 0.001, respectively) (Table 1).

**Table 1:** Demographic characteristics of the RM patients and the controls*.

	RM patients(102)	Controls(162)	*P-values*
**Age**	28.36±3.75	28.04±3.68	0.351
**No.of RM**	2.47±0.73	-	
**BMI**	21.18±2.29	20.19±2.37	**<0.001**
	n (%)	n (%)	
**Age**			
<25	18(17.65)	25(15.43)	0.256
25~29	43(42.16)	85(52.47)	
>29	41(40.20)	52(32.10)	
**BMI (kg/m** ^**2**^ **)**			
<18.5	11(10.78)	36(22.22)	**0.047**
18.5~	78(76.47)	116(71.60)	
24~	12(11.76)	9(5.56)	
≥28	1(0.98)	1(0.62)	
**Years of education**			
≤6	23(22.55)	25(15.43)	**0.001**
7~9	24(23.53)	58(35.80)	
10~12	23(22.55)	56(34.57)	
>12	32(31.37)	23(14.20)	
**Occupation**			
Workers	15(14.71)	26(16.05)	0.593
Business/services	31(30.39)	57(35.19)	
Professionals	24(23.53)	25(15.43)	
Unemployed	23(22.55)	39(24.07)	
Other	9(8.82)	4(9.26)	

*The values for age, number of RM and BMI are expressed as the mean ± SD. BMI = body mass index (kg/m^2^).

### Urinary BPA concentration in RM patients and controls

**Fig 2:**
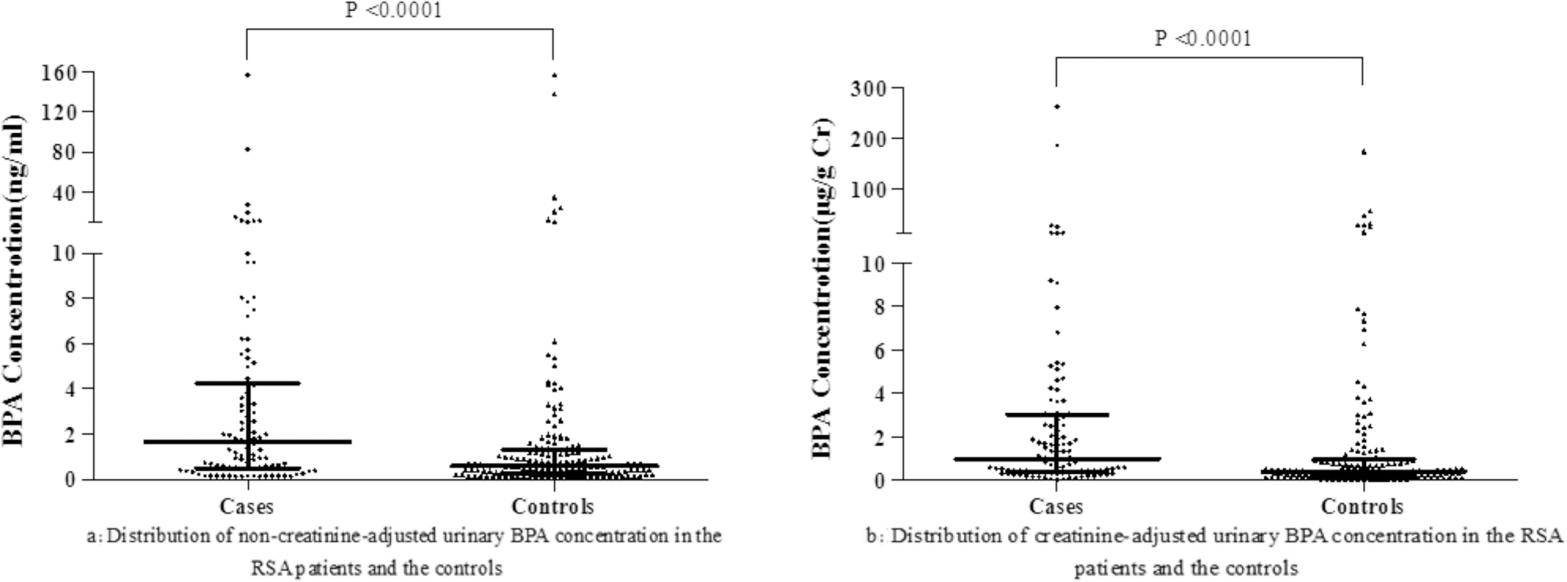
Distribution of non-creatinine-adjusted (a) and creatinine-adjusted (b) urinary BPA concentration in the RM patients and the controls.

Fig 2 shows the distributions of non-creatinine-adjusted and creatinine-adjusted urinary BPA in the RM patients and the controls. No significant differences in the detection rate of urinary BPA were found between the RM patients (85.29%) and the controls (82.10%) (*P* = 0.498). Without adjusting for creatinine, the median ± *IQR* value of total urinary BPA levels in the RM patients was 1.66±3.69ng/ml, significantly higher than the level measured in the controls (0.58±1.07ng/ml) (*P*<0.001). After adjusting for creatinine, the median ± quartile range urinary BPA level among the RM patients was 0.98±2.67μg/g Cr, significantly higher than the level among the controls (0.40±0.77μg/g Cr) (*P*<0.001). Significant differences can be observed between cases and controls in the subgroups defined by age, BMI, years of education, occupation and passive smoking during pregnancy (*P*<0.05). However, for the same RM patients group or the same controls group, we did not find that the age, BMI, years of education, occupation and passive smoking status significantly were associated with the creatinine-adjusted BPA level (all *P*>0.05) (Table 2).

**Table 2:** Creatinine-adjusted urinary BPA concentration in the RM patients and the controls (μg/g Cr).

	RM patients(102)		Controls(162)		*P-values*
	n(Detection rate)*	Median (*P*_25_-*P*_75_) (μg/g Cr)	n(Detection rate) *	Median(*P*_25_-*P*_75_) (μg/g Cr)	
**Age Age**(years)					
<25	18(88.89)	0.55(0.32–4.17)	25(88.00)	0.47(0.26–1.44)	0.242
25~29	43(86.05)	1.74(0.24–4.22)	85(84.71)	0.40(0.16–0.97)	**0.001**
>29	41(82.93)	0.85(0.43–1.65)	52(75.00)	0.37(0.14–0.73)	**0.001**
*P-values*		0.216		0.311	
**BMI**					
<18.5	11(81.82)	0.51(0.19–3.61)	36(75.00)	0.37(0.14–0.82)	0.156
18.5~	78(85.90)	1.19(0.35–3.02)	116(85.34)	0.46(0.17–0.97)	**<0.001**
24~31	13(84.62)	0.95(0.44–1.66)	10(70.00)	0.21(0.16–0.52)	**0.017**
*P-values*		0.899		0.557	
**Years of education**					
≤6	23(91.30)	1.12(0.42–2.49)	25(88.00)	0.46(0.26–0.88)	**0.041**
7~9	24(83.33)	1.63(0.33–3.89)	58(79.31)	0.39(0.14–0.73)	**0.002**
10~12	23(86.96)	1.33(0.44–5.34)	56(85.71)	0.52(0.21–1.60)	0.050
>12	32(81.25)	0.72(0.29–2.13)	23(73.91)	0.30(0.13–0.57)	**0.015**
*P-values*		0.491		0.136	
**Occupation**					
Workers	15(93.33)	0.55(0.41–5.25)	26(69.23)	0.38(0.16–0.75)	**0.021**
Business/services	31(90.32)	1.33(0.34–3.61)	57(84.21)	0.50(0.14–0.97)	**0.014**
Professionals	24(83.33)	0.90(0.31–2.19)	25(76.00)	0.38(0.14–0.90)	**0.027**
Unemployed	23(73.91)	0.54(0.32–2.49)	39(94.87)	0.40(0.21–0.73)	0.080
Other	9(88.89)	1.66(0.60–4.69)	15(73.33)	0.32(0.17–2.26)	0.190
*P-values*		0.767		0.970	
**Passive Smoking**					
No	76(82.89)	1.23(0.40–3.05)	128(82.81)	0.39(0.16–0.80)	**<0.001**
Yes	26(92.31)	0.55(0.34–2.80)	34(79.41)	0.42(0.17–1.55)	0.236
*P-values*		0.348		0.438	
**Total**	102(85.29)	0.98(0.35–3.02)	162(82.10)	0.40(0.16–0.93)	**<0.001**
		1.66(0.49–4.18) ^#^		0.58(0.24–1.31) ^#^	**<0.001**

* n = detection of total number.

^#^ The median ± IQR values of non-creatinine-adjusted total urinary BPA levels in the RM patients and the controls

### Association between urinary BPA level and RM

In the model, BPA levels were categorized into four groups: ≤*P*_*2*5_, *P*_*25*_-≤*P*_*50*_, *P*_*50*_-*P*_*75*_, >*P*_*75*_ of the control group BPA concentration. After adjusting for occupation, years of education, BMI, passive smoking during pregnancy, higher urinary BPA levels were associated with an increased risk of RM (*P*-trend<0.001). Compared to the group with urinary BPA level≤0.16μg/g Cr, the RM odds ratios for the groups with BPA levels of 0.16–0.40μg/g Cr, 0.40–0.93μg/g Cr and >0.93μg/g Cr were 2.90 (95%CI: 0.93–9.03), 3.91(95%CI:1.23–12.45), and 9.34 (95%CI: 3.06–28.44), respectively (Table 3). In addition, though significant difference for creatinine-adjusted BPA level was found between three kinds of women with 0, 2 times and >2 times RM history (*P*<0.001), no dose-response relationship existed between the RM times and BPA levels (Table 4).

**Table 3:** Association between urinary BPA level and RM.

Urinary BPA Concentration (μg/g Cr)	RM patients (%)	Controls(%)	OR(95%CI)	OR* (95%CI)	*P-values*
≤0.16	7(6.86)	41(25.31)	1.0	1.0	
0.16~	20(19.61)	40(24.69)	3.10(1.07–9.01)	2.90(0.93–9.03)	0.067
0.40~	22(21.57)	41(25.31)	3.71(1.27–10.85)	3.91(1.23–12.45)	**0.021**
>0.93	53(51.96)	40(24.69)	9.64(3.35–27.74)	9.34(3.06–28.44)	**<0.001**
Trend test					**<0.001**
age				2.25(0.97–1.62)	0.085
occupation,				1.03(0.82–1.29)	0.791
years of education				1.22(0.89–1.68)	0.213
BMI				1.23(1.05–1.44)	**0.010**
passive smoking				2.98(0.73–12.21)	0.130

*Adjusted for age, occupation, years of education, BMI, passive smoking during pregnancy

**Table 4:** Distribution of urinary BPA levels in controls, two and three or more RM patients.

	n(Detection rate)*	Median(P25-P75) (ng/ml)	Median(*P*_*25*_-*P*_*75*_)^†^ (μg/g Cr)	*F*-value	*P*-*value* ^#^
Controls	162(82.10)	0.58(0.24–1.31)^b^	0.40(0.16–0.93) ^b^	12.46	**<0.001**
Two RMs	64(90.63)	2.01(0.57–5.26)^a^	1.35(0.43–3.35) ^a^		
≥Three RMs	38(76.32)	0.95(0.22–2.38) ^b^	0.60(0.32–1.97) ^a^		

* n = detection of total number

^**†**^ Adjustment for creatinine-adjusted BPA

^#^ for creatinine-adjusted BPA

a, b: In the method of SNK analysis, there was not statistically significant difference with the same letters (a, b) (*P*>0.05); There was statistically significant difference with the different letters (a, b) (*P* <0.05).

### Association between time from recently RM date to recruitment and urinary BPA level

There was no significant difference for the median creatinine-adjusted urinary BPA concentration (μg/g Cr) among the different groups of time from recently RM date to recruitment (Wilcoxon χ^2^ = 6.48, *P* = 0.090) (Table 5). No significant rank correlation was found between the duration from RM date to recruitment and urinary BPA level (Spearman correlation coefficient r_s_ = -0.081, *P* = 0.421).

**Table 5:** Time from recently RM date to recruitment and creatinine-adjusted urinary BPA concentration (μg/g Cr)*.

Time from RM date to recruitment (days)	Mean±SD(n)	Median (*P*_25_-*P*_75_)	Min	Max
≤5	11.93±51.29(26)	0.45(0.32,1.66)	0.10	262.46
6~18	10.42±36.80(25)	1.15(0.51,3.68)	0.26	185.26
19~114	1.50±1.76(26)	0.78(0.32,1.88)	0.02	6.79
115~352	3.09±3.48(25)	1.69(0.72,4.22)	0.05	10.93

* Wilcoxon χ^2^ = 6.48, *P* = 0.090

## Discussion

In the present study, we found that the urinary BPA level of women who have experienced RM was significantly higher than the level measured in controls. The higher the urinary BPA levels of the women were, the stronger RM risk of 3~9 times was suggested. The time from recently RM date to recruitment does not influence the urinary BPA level, which has not been reported previously. The detection rates of urinary BPA reported in other countries range from 52% to 100% [38]. We found a reasonably similar detection rate in our study. The BPA level in our study was comparable to three previous studies [35, 39, 40] that reported median urinary BPA concentrations of 1.2μg/l, 1.3μg/l, and 1.2μg/l, respectively. After adjusting for creatinine, the BPA level was a little lower than two previous studies [31, 40] that reported median BPA levels of 1.7μg/g Cr and 2.2μg/g Cr, respectively. One study of a Chinese population without occupational exposure found a median urinary BPA concentration level below the LOD (0.31μg/l) [32]; the median urinary BPA of the controls in our study was 0.58ng/ml, slightly higher than the BPA level He reported. However, exposure to BPA is related to the changes in lifestyle in recent years. Thus, the exposure level to BPA may be different in different regions and over time [41–43].

To the best of our knowledge, this is the first epidemiologic study to investigate the association between urinary BPA level and RM risk in humans using a relatively large sample. The strengths of our methods also include the addition of an internal standard (d16-BPA), the relatively low limit of BPA detection and the adjustment of urinary BPA concentration by creatinine. In addition, we controlled the BMI variable in the logistic model, which was reported to be significantly associated with RM risk [44, 45].

Through two studies have been reported the association between the BPA exposure and miscarriage in women, the conclusion is inconsistent [22, 23]. To date, only Sugiura-Ogasawara and colleagues [11] have examined serum BPA levels using ELISA in 45 patients with a history of three or more (3–11) consecutive first-trimester miscarriages and 32 healthy women who had never given birth and had no history of infertility. The mean ± SD level of BPA in the patients was 2.59±5.23ng/ml, significantly higher than the level found in the controls (0.77±0.38ng/ml) (*P* = 0.024). This study suggested that high exposure to BPA may be associated with RM. Our results provided a different exposure biomarker in support of this conclusion.

Our findings were supported from animal and other human studies. It is clear that BPA has adverse effects on reproduction for animals in vitro and in vivo [17, 46, 47]. Lemos and colleagues showed that the success to achieve pregnancy decreased slightly with BPA concentration increasing in the terrestrial isopod Porcellio scaber, and there were 20% more miscarriages after exposure to 10 and 1000 mg BPA kg-1 dry soil [48]. In addition, an important study from China [19] found that zebrafish embryos exposed to BPA in early embryonic development exhibited higher levels of bioaccumulation and toxicity of BPA than in late embryonic development; they were also more likely to experience lethal or sublethal toxicity. For humans, BPA is a well- studied, well characterized, ubiquitous EDC. Previous studies demonstrated that serum BPA levels were associated with endometrial hyperplasia and ovarian dysfunction in females [27–29]. Balakrishnan and colleagues have demonstrated that BPA at low environmentally relevant levels can transfer across the human placenta [49]. What’s more, Nishikawa found that BPA‑GA is transferred into the fetus and deconjugated in the fetus [50]. There was a particularly worrying finding that human fetuses have also been contaminated with BPA [51, 52]. Serum BPA levels among women carrying fetuses with an abnormal karyotype were higher than the levels in women carrying fetuses with a normal karyotype [53]. In addition, a study found that there was a positive linear dose-response association between urinary BPA concentrations and implantation failure among women undergoing in vitro fertilization [54].

The major limitation of our study is that the BPA measurements were based on a spot urine sample. We could not acquire samples before or during the RM patients’ pregnancies, although this could have provided information about women’s daily BPA exposure during the critical period. Urinary BPA concentration has a moderate degree of intra-individual variability, making it difficult to accurately characterize exposure from a single measurement [31]. Braun et al. also mentioned that more than one sample may be necessary for BPA during pregnancy [55]. However, it has been reported that a single sample is predictive of BPA exposure over weeks or months, and a single sample can provide good sensitivity to classify a person’s exposure in epidemiologic studies [34, 56, 57]. Therefore, the urine samples we collected should be partly representative of the daily BPA exposure level of the subjects. An additional limitation is that the cases were not pregnant at the time of urine sample collection while the controls were. It cannot be excluded that the differences in BPA concentration between patients and controls could partly be due to one group being pregnant and the other not. There is recent evidence showing that serial urine concentrations of BPA were highly variable before and during pregnancy, these pregnancy induced changes may influence the absorption, distribution, metabolism, or excretion of BPA [55]. But, due to the limitation of the field conditions, we had difficulty to collect urine sample from the cases before or at the time of miscarriage. To resolve this problem, we consider to design a cohort study and to collect specimens according to the monitoring data in the future research. In this study, although we did not collect information about women chromosome abnormality and maternal endocrinologic disorders, because there is no evidence that the BPA level is associated with chromosome abnormalities and endocrine disorders, if the cases included a little number of such women, it would have underestimated the OR between the exposure and outcome. Calafat reported that BPA was associated with the use of medical devices [58]. Hence, our evidence need further studies in consideration of the medical devices, though according to our investigation of gynaecologists and obstetricians, most subjects who visited hospitals for RM were generally not treated with intravenous or intramuscular injections; instead, they were prescribed traditional Chinese medicine remedies for improving their endocrine secretion.

The biological mechanism of our findings and the above results of animals and human studies may be relevant to the chemical structure of BPA. It is similar to that of endogenous estrogens and can mimic the function of 17β-estradiol (E2) by combining with the estrogen receptors. So BPA can interfere with normal levels of hormones in the blood and functioning of estrogens, and further affect the reproductive development [55].

In conclusion, higher daily exposure to BPA was associated with an increased risk of RM, and that longitudinal studies are required to investigate the association between maternal BPA levels during pregnancy and RM.

## References

[1] Weiss A, Shalev E, Romano S. Hysteroscopy may be justified after two miscarriages. Human reproduction. 2005;20(9):2628–31. 15890730 10.1093/humrep/dei081

[2] Daya S. Evaluation and management of recurrent spontaneous abortion. Current Opinion in Obstetrics and Gynecology. 1996;8(3):188–92. 8818529

[3] Tulppala M, Palosuo T, Ramsay T, Miettinen A, Salonen R, Ylikorkala O. A prospective study of 63 couples with a history of recurrent spontaneous abortion: contributing factors and outcome of subsequent pregnancies. Human reproduction. 1993;8(5):764–70. 8314975 10.1093/oxfordjournals.humrep.a138137

[4] Li T, Makris M, Tomsu M, Tuckerman E, Laird S. Recurrent miscarriage: aetiology, management and prognosis. Human reproduction update. 2002;8(5):463–81. 12398226 10.1093/humupd/8.5.463

[5] Regan L, Owen EJ, Jacobs HS. Hypersecretion of luteinising hormone, infertility, and miscarriage. The Lancet. 1990;336(8724):1141–4. 1978024 10.1016/0140-6736(90)92765-a

[6] Stirrat G. Recurrent miscarriage I: definition and epidemiology. The Lancet. 1990;336(8716):673–5. 1975862 10.1016/0140-6736(90)92159-f

[7] Clifford K, Rai R, Watson H, Regan L. Pregnancy: An informative protocol for the investigation of recurrent miscarriage: preliminary experience of 500 consecutive cases. Human reproduction. 1994;9(7):1328–32. 7962442 10.1093/oxfordjournals.humrep.a138703

[8] Daya S. Issues in the etiology of recurrent spontaneous abortion. Current Opinion in Obstetrics and Gynecology. 1994;6(2):153–9. 8193255

[9] Greaves M, Cohen H, MacHin S, Mackie I. Guidelines on the investigation and management of the antiphospholipid syndrome. British journal of haematology. 2000;109(4):704–15. 10929019 10.1046/j.1365-2141.2000.02069.x

[10] Homer HA, Li T-C, Cooke ID. The septate uterus: a review of management and reproductive outcome. Fertility and sterility. 2000;73(1):1–14. 10632403 10.1016/s0015-0282(99)00480-x

[11] Sugiura-Ogasawara M, Ozaki Y, Sonta S-i, Makino T, Suzumori K. Exposure to bisphenol A is associated with recurrent miscarriage. Human reproduction. 2005;20(8):2325–9. 15947000 10.1093/humrep/deh888

[12] Sugiura-Ogasawara M, Ozaki Y, Sonta S-i, Makino T, Suzumori K. PCBs, hexachlorobenzene and DDE are not associated with recurrent miscarriage. American Journal of Reproductive Immunology. 2003;50(6):485–9. 14750556 10.1046/j.8755-8920.2003.00106.x

[13] Kang J-H, Kito K, Kondo F. Factors influencing the migration of bisphenol A from cans. Journal of Food Protection. 2003;66(8):1444–7. 12929833 10.4315/0362-028x-66.8.1444

[14] Le HH, Carlson EM, Chua JP, Belcher SM. Bisphenol A is released from polycarbonate drinking bottles and mimics the neurotoxic actions of estrogen in developing cerebellar neurons. Toxicology letters. 2008;176(2):149 18155859 10.1016/j.toxlet.2007.11.001 PMC2254523

[15] Kubwabo C, Kosarac I, Stewart B, Gauthier B, Lalonde K, Lalonde P. Migration of bisphenol A from plastic baby bottles, baby bottle liners and reusable polycarbonate drinking bottles. Food Additives and Contaminants. 2009;26(6):928–37. 10.1080/02652030802706725 19680968

[16] Watts MM, Pascoe D, Carroll K. Chronic exposure to 17α-ethinylestradiol and bisphenol A-effects on development and reproduction in the freshwater invertebrate Chironomus riparius(Diptera: Chironomidae). Aquatic Toxicology. 2001;55(1):113–24. 11551626 10.1016/s0166-445x(01)00148-5

[17] Honma S, Suzuki A, Buchanan DL, Katsu Y, Watanabe H, Iguchi T. Low dose effect of in utero exposure to bisphenol A and diethylstilbestrol on female mouse reproduction. Reproductive toxicology (Elmsford, NY). 2002;16(2):117 11955942 10.1016/s0890-6238(02)00006-0

[18] Herath CB, Jin W, Watanabe G, Arai K, Suzuki AK, Kazuyoshi Taya D. Adverse effects of environmental toxicants, octylphenol and bisphenol A, on male reproductive functions in pubertal rats. Endocrine. 2004;25(2):163–72. 15711031 10.1385/ENDO:25:2:163

[19] Duan Z-H, Zhang B-T, Zhu L. The toxicity and bioaccumulation of bisphenol A on developmental stages of zebrafish embryo China Environmental Scienc. 2008;3:018.

[20] Zhang H-Q, Zhang X-F, Zhang L-J, Chao H-H, Pan B, Feng Y-M, et al Fetal exposure to bisphenol A affects the primordial follicle formation by inhibiting the meiotic progression of oocytes. Molecular biology reports. 2012;39(5):5651–7. 10.1007/s11033-011-1372-3 22187349

[21] Chao H-H, Zhang X-F, Chen B, Pan B, Zhang L-J, Li L, et al Bisphenol A exposure modifies methylation of imprinted genes in mouse oocytes via the estrogen receptor signaling pathway. Histochemistry and cell biology. 2012;137(2):249–59. 10.1007/s00418-011-0894-z 22131059

[22] Lathi RB, Liebert CA, Brookfield KF, Taylor JA, vom Saal FS, Fujimoto VY, et al Conjugated bisphenol A in maternal serum in relation to miscarriage risk. Fertility and sterility. 2014;102(1):123–8. 10.1016/j.fertnstert.2014.03.024 . 24746738 PMC4711263

[23] Chen X, Chen M, Xu B, Tang R, Han X, Qin Y, et al Parental phenols exposure and spontaneous abortion in Chinese population residing in the middle and lower reaches of the Yangtze River. Chemosphere. 2013;93(2):217–22. 10.1016/j.chemosphere.2013.04.067 23714150

[24] Meeker JD, Ehrlich S, Toth TL, Wright DL, Calafat AM, Trisini AT, et al Semen quality and sperm DNA damage in relation to urinary bisphenol A among men from an infertility clinic. Reproductive toxicology. 2010;30(4):532–9. 10.1016/j.reprotox.2010.07.005 20656017 PMC2993767

[25] Li D, Zhou Z, Qing D, He Y, Wu T, Miao M, et al Occupational exposure to bisphenol-A (BPA) and the risk of self-reported male sexual dysfunction. Human reproduction. 2010;25(2):519–27. 10.1093/humrep/dep381 19906654

[26] Bloom MS, vom Saal FS, Kim D, Taylor JA, Lamb JD, Fujimoto VY. Serum unconjugated bisphenol A concentrations in men may influence embryo quality indicators during in vitro fertilization. Environmental toxicology and pharmacology. 2011;32(2):319–23. 10.1016/j.etap.2011.06.003 21843814 PMC3157013

[27] Hiroi H, Tsutsumi O, Takeuchi T, Momoeda M, Ikezuki Y, Okamura A, et al Differences in serum bisphenol a concentrations in premenopausal normal women and women with endometrial hyperplasia. Endocrine journal. 2004;51(6):595 15644579 10.1507/endocrj.51.595

[28] Takeuchi T, Tsutsumi O, Ikezuki Y, Takai Y, Taketani Y. Positive relationship between androgen and the endocrine disruptor, bisphenol A, in normal women and women with ovarian dysfunction. Endocrine journal. 2004;51(2):165 15118266 10.1507/endocrj.51.165

[29] Politch JA. Bisphenol A and risk assessment. Environmental Health Perspectives. 2006;114(1):A16 16393639 10.1289/ehp.114-a16a PMC1332690

[30] Völkel W, Colnot T, Csanády GA, Filser JG, Dekant W. Metabolism and kinetics of bisphenol A in humans at low doses following oral administration. Chemical research in toxicology. 2002;15(10):1281–7. 12387626 10.1021/tx025548t

[31] Braun JM, Yolton K, Dietrich KN, Hornung R, Ye X, Calafat AM, et al Prenatal bisphenol A exposure and early childhood behavior. Environmental Health Perspectives. 2009;117(12):1945 10.1289/ehp.0900979 20049216 PMC2799471

[32] He Y, Miao M, Herrinton LJ, Wu C, Yuan W, Zhou Z, et al Bisphenol A levels in blood and urine in a Chinese population and the personal factors affecting the levels. Environmental research. 2009;109(5):629–33. 10.1016/j.envres.2009.04.003 19426969

[33] Matsumoto A, Kunugita N, Kitagawa K, Isse T, Oyama T, Foureman GL, et al Bisphenol A levels in human urine. Environmental Health Perspectives. 2003;111(1):101 12515686 10.1289/ehp.5512 PMC1241312

[34] García-Prieto A, Lunar ML, Rubio S, Pérez-Bendito D. Determination of urinary bisphenol A by coacervative microextraction and liquid chromatography-fluorescence detection. Analytica chimica acta. 2008;630(1):19–27. 10.1016/j.aca.2008.09.060 19068322

[35] Ye X, Pierik FH, Hauser R, Duty S, Angerer J, Park MM, et al Urinary metabolite concentrations of organophosphorous pesticides, bisphenol A, and phthalates among pregnant women in Rotterdam, the Netherlands: the Generation R study. Environmental research. 2008;108(2):260 10.1016/j.envres.2008.07.014 18774129 PMC2628162

[36] Cantonwine D, Meeker JD, Hu H, Sánchez BN, Lamadrid-Figueroa H, Mercado-García A, et al Bisphenol a exposure in Mexico City and risk of prematurity: a pilot nested case control study. Environmental Health. 2010;9:62 10.1186/1476-069X-9-62 20955576 PMC2965706

[37] Hornung RW, Reed LD. Estimation of average concentration in the presence of nondetectable values. Applied occupational and environmental hygiene. 1990;5(1):46–51.

[38] Vandenberg LN, Hauser R, Marcus M, Olea N, Welshons WV. Human exposure to bisphenol A (BPA). Reproductive Toxicology. 2007;24(2):139–77. 17825522 10.1016/j.reprotox.2007.07.010

[39] Ouchi K, Watanabe S. Measurement of bisphenol A in human urine using liquid chromatography with multi-channel coulometric electrochemical detection. Journal of Chromatography B. 2002;780(2):365–70. 12401363 10.1016/s1570-0232(02)00547-0

[40] Wolff MS, Engel SM, Berkowitz GS, Ye X, Silva MJ, Zhu C, et al Prenatal phenol and phthalate exposures and birth outcomes. Environmental health perspectives. 2008;116(8):1092 10.1289/ehp.11007 18709157 PMC2516577

[41] Yang M, Kim S-Y, Lee S-M, Chang S-S, Kawamoto T, Jang J-Y, et al Biological monitoring of bisphenol A in a Korean population. Archives of environmental contamination and toxicology. 2003;44(4):0546–51. 10.1007/s00244-002-2124-0 12712285

[42] Lang IA, Galloway TS, Scarlett A, Henley WE, Depledge M, Wallace RB, et al Association of urinary bisphenol A concentration with medical disorders and laboratory abnormalities in adults. JAMA: the journal of the American Medical Association. 2008;300(11):1303–10. 10.1001/jama.300.11.1303 18799442

[43] LaKind JS, Levesque J, Dumas P, Bryan S, Clarke J, Naiman DQ. Comparing United States and Canadian population exposures from National Biomonitoring Surveys: Bisphenol A intake as a case study. Journal of Exposure Science and Environmental Epidemiology. 2012;22(3):219–26. 10.1038/jes.2012.1 22333730 PMC3331622

[44] Wang JX, Davies MJ, Norman RJ. Obesity increases the risk of spontaneous abortion during infertility treatment. Obesity research. 2002;10(6):551–4. 12055331 10.1038/oby.2002.74

[45] Bellver J, Rossal LP, Bosch E, Zúñiga A, Corona JT, Meléndez F, et al Obesity and the risk of spontaneous abortion after oocyte donation. Fertility and sterility. 2003;79(5):1136–40. 12738508 10.1016/s0015-0282(03)00176-6

[46] Richter CA, Birnbaum LS, Farabollini F, Newbold RR, Rubin BS, Talsness CE, et al In vivo effects of bisphenol A in laboratory rodent studies. Reproductive toxicology (Elmsford, NY). 2007;24(2):199 17683900 10.1016/j.reprotox.2007.06.004 PMC2151845

[47] Wetherill YB, Akingbemi BT, Kanno J, McLachlan JA, Nadal A, Sonnenschein C, et al In vitro molecular mechanisms of bisphenol A action. Reproductive Toxicology. 2007;24(2):178–98. 17628395 10.1016/j.reprotox.2007.05.010

[48] Lemos M, Van Gestel C, Soares A. Reproductive toxicity of the endocrine disrupters vinclozolin and bisphenol A in the terrestrial isopod Porcellio scaber(Latreille, 1804). Chemosphere. 2010;78(7):907–13. 10.1016/j.chemosphere.2009.10.063 20015537

[49] Balakrishnan B, Henare K, Thorstensen EB, Ponnampalam AP, Mitchell MD. Transfer of bisphenol A across the human placenta. American journal of obstetrics and gynecology. 2010;202(4):393 e1-. e7. 10.1016/j.ajog.2010.01.025 20350650

[50] Nishikawa M, Iwano H, Yanagisawa R, Koike N, Inoue H, Yokota H. Placental transfer of conjugated bisphenol A and subsequent reactivation in the rat fetus. Environmental health perspectives. 2010;118(9):1196 10.1289/ehp.0901575 20382578 PMC2944077

[51] Sakurai K, Mori C. Fetal exposure to endocrine disruptors]. Nihon rinsho Japanese journal of clinical medicine. 2000;58(12):2508 11187746

[52] Brock JW, Yoshimura Y, Barr JR, Maggio VL, Graiser SR, Nakazawa H, et al Measurement of bisphenol A levels in human urine. Journal of exposure analysis and environmental epidemiology. 2001;11(4):323 11571611 10.1038/sj.jea.7500174

[53] Yamada H, Furuta I, Kato EH, Kataoka S, Usuki Y, Kobashi G, et al Maternal serum and amniotic fluid bisphenol A concentrations in the early second trimester. Reproductive Toxicology. 2002;16(6):735–9. 12401500 10.1016/s0890-6238(02)00051-5

[54] Ehrlich S, Williams PL, Missmer SA, Flaws JA, Berry KF, Calafat AM, et al Urinary bisphenol A concentrations and implantation failure among women undergoing in vitro fertilization. Environmental health perspectives. 2012;120(7):978 10.1289/ehp.1104307 22484414 PMC3404656

[55] Braun JM, Smith KW, Williams PL, Calafat AM, Berry K, Ehrlich S, et al Variability of urinary phthalate metabolite and bisphenol A concentrations before and during pregnancy. Environmental health perspectives. 2012;120(5):739 10.1289/ehp.1104139 22262702 PMC3346778

[56] Mahalingaiah S, Meeker JD, Pearson KR, Calafat AM, Ye X, Petrozza J, et al Temporal variability and predictors of urinary bisphenol A concentrations in men and women. Environmental Health Perspectives. 2008;116(2):173 10.1289/ehp.10605 18288314 PMC2235217

[57] Teitelbaum S, Britton J, Calafat A, Ye X, Silva M, Reidy J, et al Temporal variability in urinary concentrations of phthalate metabolites, phytoestrogens and phenols among minority children in the United States. Environmental research. 2008;106(2):257–69. 17976571 10.1016/j.envres.2007.09.010

[58] Calafat AM, Weuve J, Ye X, Jia LT, Hu H, Ringer S, et al Exposure to bisphenol A and other phenols in neonatal intensive care unit premature infants. Environmental Health Perspectives. 2009;117(4):639 10.1289/ehp.0800265 19440505 PMC2679610

